# Pursuing Sleep Architecture Remodeling: Effects of Psychopharmaceuticals on Sleep Structure

**DOI:** 10.1192/j.eurpsy.2024.655

**Published:** 2024-08-27

**Authors:** J. Camilo

**Affiliations:** Psychiatry, CHTMAD, Vila Real, Portugal

## Abstract

**Introduction:**

Sleep plays a pivotal role in overall physical and mental health, exerting a profound influence on general well-being and quality of life. The influence of psychopharmaceuticals on sleep structure is a critical research area, given their widespread use in the treatment of psychiatric disorders, yet their precise effects on sleep remain inadequately understood.

**Objectives:**

This study aims to investigate how psychopharmaceuticals affect sleep architecture by identifying commonalities and disparities among different classes of psychotropic medications.

**Methods:**

Systematic review of the literature encompassing studies assessing the effects of psychopharmaceuticals on sleep structure. Electronic databases such as PubMed were employed to identify pertinent studies published within the last decade.

**Results:**

Diverse classes of psychopharmaceuticals have varying effects on sleep architecture. Additionally, prolonged use of specific psychopharmaceuticals was correlated with sleep disturbances, such as insomnia. These findings hold significant implications for clinical practice, emphasizing the necessity of an individualized approach in treating patients with psychiatric disorders.

**Conclusions:**

Psychopharmaceuticals exert a substantial impact on sleep architecture, with effects contingent on drug class and duration of use. Understanding these alterations is crucial for optimizing the treatment of patients with psychiatric disorders, striking a balance between therapeutic benefits and potential sleep-related adverse effects. Furthermore, these discoveries underscore the importance of closely monitoring the sleep of patients undergoing psychopharmacological treatment and tailoring therapeutic approaches in accordance with individual needs.

**Disclosure of Interest:**

None Declared

